# A Nation of Gluttons

**Published:** 1884-07

**Authors:** 


### A NATION OF GLUTTONS.

"THIS is a nation of gluttons." The speaker was the famous Dr. Tanner. "If people would eat and drink less there would be no more use for doctors. They could all shut up and go off on a prolonged picnic."

"They would probably object to that."

"Of course."

"Of what practical benefit do you consider your great fast has been?"

"It has been of benefit in various ways. It has turned the attention of the people, as well as the physicians, to the efficacy of fasting as a cure for diseases of all kinds ; which I contend it is. In my own case, for instance. I formerly had heart disease so bad that competent physicians pronounced it incurable. Since my fast I have had no trace of it whatever. The fast was published and talked about all over the country, and set people to thinking. Now many, instead of taking nauseous medicine for sickness simply go without food four or five days and find themselves cured. I meet men frequently who say to me that after reading of the results of my fast and what I said, they adopted that plan for the cure of sickness, and now they have no use for physicians. Is not the theory reasonable ? Nature takes away a man's appetite when he is sick, evidently intending that he shall not eat, and thus give his digestive organs a chance to recuperate."

